# Effectiveness of Prevailing Flush Guidelines to Prevent Exposure to Lead in Tap Water

**DOI:** 10.3390/ijerph15071537

**Published:** 2018-07-20

**Authors:** Adrienne Katner, Kelsey Pieper, Komal Brown, Hui-Yi Lin, Jeffrey Parks, Xinnan Wang, Chih-Yang Hu, Sheldon Masters, Howard Mielke, Marc Edwards

**Affiliations:** 1 School of Public Health, Louisiana State University Health Sciences Center, New Orleans, LA 70112, USA; kokomole786@yahoo.com (K.B.); hlin1@lsuhsc.edu (H.-Y.L.); xwang3@lsuhsc.edu (X.W.); chu@lsuhsc.edu (C.-Y.H.); 2 Department of Civil and Environmental Engineering, Virginia Tech, Blacksburg, VA 24061, USA; kpieper@vt.edu (K.P.); Jparks@vt.edu (J.P.); edwardsm@vt.edu (M.E.); 3 Corona Environmental Consulting, Philadelphia, PA 19146, USA; smasters@coronaenv.com; 4 Department of Pharmacology, School of Medicine, Tulane University, New Orleans, LA 70112, USA; hmielke@tulane.edu

**Keywords:** drinking water, lead, Pb, flush, exposure prevention, intervention, lead service line

## Abstract

Flushing tap water is promoted as a low cost approach to reducing water lead exposures. This study evaluated lead reduction when prevailing flush guidelines (30 s–2 min) are implemented in a city compliant with lead-associated water regulations (New Orleans, LA, USA). Water samples (*n* = 1497) collected from a convenience sample of 376 residential sites (2015–2017) were analyzed for lead. Samples were collected at (1) first draw (*n* = 375) and after incremental flushes of (2) 30–45 s (*n* = 375); (3) 2.5–3 min (*n* = 373), and (4) 5.5–6 min (*n* = 218). There was a small but significant increase in water lead after the 30 s flush (vs. first draw lead). There was no significant lead reduction until the 6 min flush (*p* < 0.05); but of these samples, 52% still had detectable lead (≥1 ppb). Older homes (pre-1950) and low occupancy sites had significantly higher water lead (*p* < 0.05). Each sample type had health-based standard exceedances in over 50% of sites sampled (max: 58 ppb). While flushing may be an effective short-term approach to remediate high lead, prevailing flush recommendations are an inconsistently effective exposure prevention measure that may inadvertently increase exposures. Public health messages should be modified to ensure appropriate application of flushing, while acknowledging its short-comings and practical limitations.

## 1. Introduction

The knowledge that no safe threshold for childhood lead (Pb) exposures has been found [1], and increased awareness of Pb in drinking water triggered by the events in Flint (MI, USA), have contributed to a renewed emphasis on preventing exposure to Pb in drinking water. Water lead can exist in sites built prior to 1986 with lead service lines (LSLs), which are often the greatest contributor to water Pb when it is present [2]. Water Pb can also come from Pb solder used in homes built before 1986, or from galvanized pipes and brass faucet fixtures/fittings through present day construction practices [3]. When these sources of Pb are present, waterborne Pb may represent the most significant source of total Pb exposure for formula-fed infants [4].

The United States (U.S.) Environmental Protection Agency’s (EPA) 1991 Lead and Copper Rule, regulates control of Pb in tap water; which mandates that water utilities collect first draw tap water samples (collected after at least a six hour period of water stagnation) in high-risk homes (i.e., homes with lead service lines, and homes with copper pipes with lead solder installed after 1982) [5]. In 2010, the EPA acknowledged that significant “exposure to <water> lead may be taking place, even though the <Pb> action level is not exceeded” [6]; and in 2015, the National Drinking Water Advisory Council re-emphasized that the Lead and Copper Rule was not intended to ensure protection of all individuals from waterborne Pb exposure—rather, it was designed as a regulatory tool to identify system wide problems and broadly reduce lead exposure [7]. One reason for these conclusions are weaknesses inherent in the regulation which allow up to 10% of water samples to have any level of lead, as long as all other samples do not exceed the Pb action level (AL) of 15 ppb. [3]. However, water lead levels (WLLs) below the drinking water Pb AL are predicted to cause exceedance of the U.S. Centers for Disease Control and Prevention’s (CDC) childhood blood lead level Reference Level [5 micrograms per deciliter (µg/dL)] in 9–25% of exposed children [8,9]; and chronic exposure to WLLs as low as 1 part per billion (ppb), which is the detection limit for many laboratories, have been estimated to increase a child’s blood lead level by 35% after 150 days [10]. This information underscores a critical need for vulnerable populations to take proactive precautionary measures to prevent chronic exposures to low-dose waterborne lead.

Flushing is a widely recommended practice to reduce consumer exposure to Pb. Studies report that repeated periods of extended flushing at high flow rates are an effective remediation strategy when there are high levels of dissolved Pb [11,12,13,14,15,16]. The Consumer Confidence Report Rule (63 FR 44511, §141.154) requires that water utilities promote flushing on all annual reports to consumers, “regardless if a system did or did not detect lead” [17]. This requirement was brought about by EPA’s recognition that even in Pb-compliant cities, “there are situations where the most vulnerable populations may be exposed to elevated levels of lead for many months before or without being notified” [17]. The EPA also requires utilities to promote flushing when a utility is not Pb-compliant. The original messaging required by the Public Education provision of the Lead and Copper Rule (56 FR 26460 §141.85) was “Run the water for 15–30 s (or one minute if the home has a lead service line), before drinking water to flush lead from interior plumbing” [5,17].

The Washington DC, USA Lead Crisis (2001–2004) first demonstrated that the standard water Pb avoidance flushing guidance was inadequate during AL exceedances, and that flushing for only 15–30 s would directly expose consumers to hazards of water that had been held within the lead service lines [15,18]. A decade of follow up research has since confirmed that flushing protocols which reduce exposure in a given home, are highly dependent on variables that are difficult or impossible to control, including, but not limited to the length, configuration, material, condition and disturbance of service lines or plumbing, water use patterns, spatial changes in chemical and microbiological water quality within a given distribution system; and type of Pb released (particulates vs. dissolved) [2,3,11,14,15,19,20,21,22,23,24,25,26,27,28,29,30,31,32,33,34,35,36,37]. In light of the evidence challenging the efficacy of flushing under different conditions, the EPA identified a need to further evaluate flushing [38] and revised the Lead and Copper Rule and Consumer Confidence Report Rule to allow utilities to modify the required flush time recommendations if they determine longer flush times are needed [17,39,40]. The EPA indicated in its updated 2010 guidance to utilities that “It is likely that systems with lead service lines will need to collect data to determine the appropriate flushing times” [6].

However, this knowledge has not translated into widespread changes in public health messaging or policies, perhaps because of the dearth of published data on flushing ineffectiveness under select conditions. Despite the new flexibility authorized by the EPA, industry knowledge of the inconsistent effectiveness of flushing, and acknowledgement by government officials about the uncertainty of optimal flush times and frequencies, officials from water utilities [41] and federal agencies [42,43,44] continued to provide outdated outreach materials with the harmful advice to flush water for 15–30 s in systems with lead service lines. While some utilities and public health officials have resorted to adding a general caveat to their risk reduction messages, that “longer flushing may be required” dependent on site specific site circumstances, that message does nothing to inform consumers when that instruction applies to their situation; and it leaves open what “longer flushing” means.

This study explicitly examines concerns that flushing may not be an effective lead reduction strategy in cities with lead service lines that are compliant with the Lead and Copper Rule. Prior research on flushing efficacy has typically been conducted in cities with non-compliant systems (i.e., high WLLs and Lead and Copper Rule AL exceedances), rather than the lower-to-moderate WLLs that are typically associated with water systems with optimized corrosion control treatment [31]. To address this gap, this study was conducted in New Orleans (LA, USA), a city which has consistently met the Lead and Copper Rule requirements. Best estimates from the mid-1990 s suggested that lead service lines may comprise 65–80% of the city’s service line system [45]. After EPA regulations on flush time recommendations were relaxed, the city’s water utility, the Sewerage and Water Board, continued to promote the original flush recommendations from 2009 to 2015 [41,46]. At the commencement of this study, the utility encouraged residents to flush their taps “for 30 s to 2 min before using water for drinking or cooking” daily under normal use conditions [41] (Figure S1, Supplementary Materials). New Orleans is also representative of many U.S. cities today, in that it has relatively non-corrosive water, and an aging drinking water infrastructure in need of repair [47]. Hurricane damage to water infrastructure has necessitated a multi-year project to repair and replace corroding water mains, lead service lines and other underground utilities throughout the city. While over ten years have passed since Hurricane Katrina, the rebuilding process is ongoing—city and utility officials are also in the process of conducting 16,000 partial lead service lines replacements [48].

The primary aim of this study is to evaluate the effectiveness of prevailing flush time recommendations commonly promoted by utilities and public health officials for New Orleans. Specifically, Pb levels were measured in cold water post-stagnation samples that were collected at first draw and after various flush times (30 s, 2.5–3 min and 5.5–6 min). A second objective of this study is to identify factors which may be associated with WLLs in an effort to better understand conditions which may contribute to high WLLs, and identify sites in potential need of targeted monitoring, outreach, or intervention. Results reveal that low-occupancy and older homes have significantly higher WLLs; and suggest that lead service lines may be a main contributor to New Orleans’ WLLs. Flushing according to prevailing guidelines (30 s to 2 min) does not result in significant or substantial water Pb reduction in the city.

## 2. Materials and Methods

### 2.1. Site Selection and Sampling Campaign

This study focused recruitment and sampling efforts in New Orleans—in particular, on the city’s East Bank of the Mississippi River (the city’s source water). Specific information about the water treatment system and water quality parameters can be found in the Supplementary Materials section (Table S1). Between February 2015 and November 2016, a convenience sample of 450 New Orleans East Bank residents were recruited via news media and word of mouth to participate in a free water testing effort. Participation entailed collecting tap water and completion of a household survey. Recruited homes met one or more of the following criteria or indicators of potential risk for inclusion in the study: (1) sites with lead service lines or galvanized pipes based on water utility data or self-reports (10%); (2) buildings constructed prior to 1950 based on self-reports an approach used by (59%) [49]; (3) homes of families with lead-poisoned children directed to the study by State’s Office of Public Health (8%); and buildings located in high risk neighborhoods, as determined by the utility’s regulatory compliance data (26%). Some sites met multiple criteria—for example, of the 8% of homes with lead-poisoned children, 76% also lived in pre-1950 homes and one reported having a lead service line.

During the study there were opportunities to evaluate WLLs in samples collected from buildings with atypical water use conditions, including unoccupied and irregular use sites (*n* = 6 unoccupied homes, *n* = 2 sample kits from 1 church, and *n* = 21 sample kits from 9 schools); and after lead service line replacements (*n* = 5). Residents in homes with LSL replacements were encouraged to purchase water filters—these results are presented in Supplementary Table S2. All data from unoccupied or irregular use sites were analyzed separately from normal use occupied homes. Other water samples were removed from the main analyses including: Samples collected incorrectly (*n* = 1); samples collected outside the area serviced by the East Bank water treatment system (*n* = 4); and samples collected from control sites (*n* = 6 sites). In all 45 water kits were removed from the main analysis.

Of the original 450 water collection kits and surveys dispensed, 421 water collection kits were returned (94% response rate, *n* = 450), and 409 surveys were returned (91% response rate, *n* = 450). Of the water collection kits returned, 45 were removed from the main analysis (for the reasons already described). In total, 376 water kits from 376 occupied normal use residences were included in the final analyses [93% response rate among those eligible for the study (*n* = 405)]. Each water kit contained 4 water collection bottles, but not all households collected each sample and some bottles leaked during transport. Each of the 376 water kits analyzed had at least 1 returned water bottle—the total number of water samples collected was 1497. Of the 376 eligible sample kits, 15 households did not return surveys, leading to a final number of 361 surveys included in the survey analyses for occupied normal use households [96% survey response rate among eligible participants returning kits (*n* = 376); and 89% among those eligible for the study (*n* = 405)]. Some survey respondents did not respond to all questions—this impacted the sample size for each question in the multivariate model. Participant and household characteristics (and associated minimum, mean, median and maximum WLLs) are presented in Table S3 (Supplementary Materials).

The study protocols and survey were reviewed and approved by the Louisiana State University Health Sciences Center Institutional Review Board (IRB) (FWA 00002762) to assure protection of human research subjects (IRB 8870). Participation in the study did not begin until a study consent had been obtained. The lengths of water service lines and premise plumbing pipes were estimated based on resident measurements reported on returned surveys (Figure S2, Supplementary Materials). Residents were asked to measure the distance from the middle of the street to the water line as it enters the home (service line length) and the distance from where line enters home to the kitchen tap as measured along wall (premise plumbing). Researchers also derived google map measurements of potential service line lengths for all sites, based on measures taken from the center of the street to the front of the home in the satellite view of Google Maps using the distance and area tool.

### 2.2. Sampling Protocol

Residents were provided with a sampling kit that contained: (1) sampling instructions; (2) four 250 mL wide-mouth sampling bottles; (3) pre-paid return postage; and (4) a questionnaire about the household and water use characteristics (see survey in Supplementary Materials). To evaluate the effectiveness of flush time recommendations, residents were instructed to collect unfiltered tap water from the kitchen sink, after a 6+ hour stagnation period. Residents were instructed not to clean or take off their aerator prior to the stagnation period or water collection. Residents were instructed to collect water at “normal to high water flow” [estimated flow rate of 3.0 to 8.3 L/minute (0.8 to 2.2 gallons/minute)] [50]. Specifically, a 250 milliliter (mL) first draw cold-water sample (FD) was collected and the water was shut off. A 250 mL first draw hot sample (FDH) was then collected from the hot water tap and immediately shut off after sample collection (the FDH water sample was not collected after the water temperature increased, rather it was collected at first draw). Two samples were collected after flushing cold water for 30–45 s (F30S), and after flushing for an additional 2 min (2.5–3 min total flushing; F3M). Throughout the entire study, all sites were asked to collect FD, F30S, and F3M samples. Mid-way through the study, it became apparent that flushing did not consistently reduce WLLs. At this point, an extended flush time sample was collected in lieu of the FDH samples. Residents were asked to flush their taps for an additional 3 min after collecting the F3M samples. These new samples (F6M) were collected after a 5.5–6 min total flush time.

### 2.3. Analytical Methods

Sampling kits were shipped by residents to Virginia Tech for analysis. Water samples were acidified with nitric acid (2% *v*/*v*) and digested for 16+ hours before analysis on a X-Series Inductively Coupled Plasma–Mass Spectrometry (ICP-MS, Thermo Electron, Waltham, MA, USA) per method 3125 B—typical recovery is 100% with a 2% nitric acid preservation and a 16+ hour holding time [51]. Our current limit of quantification is 1 ppb. Blanks and/or spikes of known concentrations were processed every 10 samples for QA/QC purposes. Blanks were below detection; and the percent relative standard deviation (%RSD) was <2%. Blind negative controls, which consisted of nine samples of filtered water from six sites, were sent to the lab to confirm laboratory reporting—all negative controls were <1 ppb. Other source-specific metals were analyzed to evaluate correlations with WLLs and identify potential Pb sources in New Orleans tap water. These included: cadmium, chromium, copper, iron, nickel, tin, and zinc.

### 2.4. Statistical Analysis

The WLLs for the FD, FDH, F30S, F3M, and F6M samples were summarized using descriptive statistics. Samples with WLLs less than 1 ppb were below our reporting limit (1 ppb) and were considered non-detects (ND). To represent ND numerically, these samples were assigned a value of half the reporting limit (0.5 ppb). The WLL differences between samples collected after various flushing times and the FD sample were tested using the Wilcoxon signed-rank test. Statistical analyses were performed in SAS Version 9.4 (SAS, Cary, NC, USA). The significance level in this study was defined as *p*-value < 0.05, unless otherwise stated.

To identify factors associated with detectable WLLs (≥1 ppb), frequencies and percentages of households with ≥1 ppb were calculated by participant and household characteristics. The candidate factors included participant characteristics (income, race and education) and household factors (number of occupants in household, number of children < 6 years old, presence or absence of street or sidewalk work within the last 6 months on site block, era of building construction (pre-, and post-1950), home type (single-family, multi-family, apartment complex), home ownership (own or rent), and water usage). Water usage, based on resident reports from last monthly utility bill (monthly total and average daily water usage), had a limited sample size (*n* = 38) so it was not included in modeling. Factors associated with detectable WLL (≥1 ppb) based on all cold water samples were analyzed using mixed-effects logistic models. In mixed models, the household providing water samples was treated as the random effect. Both univariate and multivariable models were conducted to evaluate the relationship between detectable WLLs and participant and household factors. Factors with a *p*-value < 0.2 in the models after adjusting for flush time were candidates for building the multivariable models. The final multivariable models only included predictors with a *p*-value < 0.05.

To evaluate WLLs in relation to health or regulatory criteria for all samples and by sample type, the percent of samples exceeding the following standards, criteria or goals was derived: (1) the American Academy of Pediatrics’ (AAP) recommended level for water in schools (AAP RL, 1 ppb) [52]; (2) the U.S. Food and Drug Administration’s (FDA) allowable lead level in bottled water (FDA AL, 5 ppb) [53]; (3) the World Health Organization’s (WHO) provisional guideline value for Pb in water (WHO GV, 10 ppb) [54], (4) the US EPA’s Pb AL (15 ppb) [5], and (5) the US EPA’s Maximum Contaminant Level Goal (MCLG, 0 ppb) for Pb, the WLL that EPA considers to be safe (US EPA 1991 [5]).

To determine the most probable location in the water distribution system or premise plumbing that each sample type may have been sitting during the stagnation period, an estimate of the volume of water and flush times required to purge the lines was derived based on estimated flow rates at low flow (3.0 L per minute) and high flow (8.3 L per minute); typical premise and service line pipe diameters; and survey respondent measurements of service lines and premise plumbing (Figure S2, Supplementary Materials). A 250-mL sample is estimated to represent water in approximately 2.4 m (or 8 feet) of piping.

## 3. Results

### 3.1. Water Lead Levels and Flushing Efficacy for Normal Use Occupied Homes

**Figure 1:**
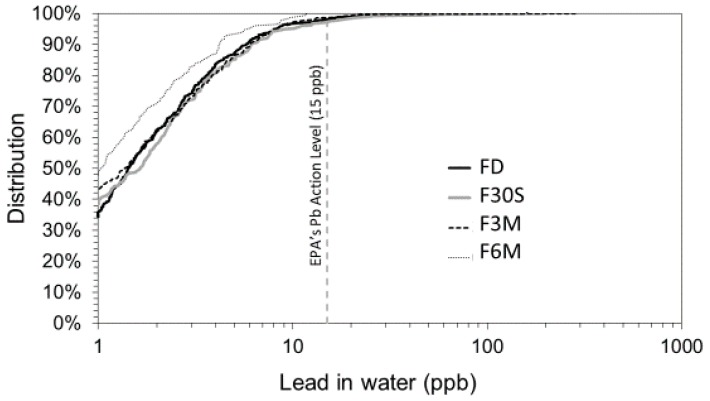
Cumulative distribution of total water lead levels (WLLs) in occupied normal-use homes by sample type (*n* = 1497 samples from 376 sites). **Key:** FD: first draw; F30S: 30–45 s total flush; F3M: 2.5–3 min total flush; F6M: 5.5–6 min total flush.

Descriptive summary statistics for WLLs from normal use occupied homes (Table 1), indicate median WLLs for the FD, F30S, F3M and F6M cold water samples of 1.4, 1.7, 1.4 and 1.1 ppb, respectively. Overall New Orleans WLLs were typically low relative to the 15 ppb EPA AL, as 88% of all samples from normal-use residential sites had WLLs ≤ 5 ppb. However, low-dose waterborne Pb exposures (≥1 ppb) are widespread across the city, as half of all samples from normal use sites (60%) had detectable WLLs of at least 1 ppb or higher (Table 1). Median and maximum WLLs were highest for post-stagnation samples collected after the 30 s flush (F30S), and lowest for post-stagnation samples collected after a 6 min total flush (F6M). There was wide variability in WLLs across the sample pool, with WLLs ranging from non-detect (<1 ppb) in each sample type to a maximum of 58 ppb in F30S samples. The cumulative distributions of total Pb concentrations by water sample type for normal use occupied homes did not change substantially between FD samples and F30S or F3M samples (Figure 1). It was not until after 6 min of flushing that a decrease in the WLL distribution was observed.

The results of the WLL differences from FD to flushed samples (Table 2, Figure S3) demonstrate a small but significant increase (median = 0 ppb and mean = 0.6 ppb, *p* = 0.04) in WLLs from FD to F30S sample. No significant change in WLLs was observed from FD to F3M samples (*p* = 0.219). Small but significant declines in WLLs was observed in F6M (median = 0 ppb and mean = −0.2 ppb) and FDH (median = −0.1 ppb and mean = −0.4 ppb) samples, compared to FD samples (Table 2; Figure S3). Even after flushing for 5.5 to 6 min, over half of F6M samples (52%, Table 1) still had detectable WLLs (≥1 ppb), while 7% had WLLs > 5 ppb (Table 5). Flushing may seems to have induced the mobilization of particulate Pb into water, as evidenced by high “spikes” in some post-flushing samples—WLLs increased to as much as 58 ppb in one single-resident home after 30 s of flushing (Table 2).

Table S4 presents the number of samples in each WLL category by sample type; and Table 3 presents the number and percent of samples with a change in WLL detection or with insubstantial WLL changes (<1 ppb), compared to FD WLLs. The majority of the households (80–81%) had no change in WLL (<1 ppb difference) in flushed samples compared to FD samples (Table 3). In general, most homes with FD WLLs below the reporting limit (<1 ppb) continued to have WLLs < 1 ppb in flushed samples. Of sites with FD WLLs < 1 ppb, 79%, 83% and 86% also had WLLs < 1 ppb in F30S samples (*n* = 136), F3M samples (*n* = 135), and F6M samples (*n* = 86), respectively (Table S4). Additionally, most homes with detectable FD WLLs (≥1 ppb) continued to have WLLs ≥ 1 ppb in flushed samples. Of sites with FD WLLS ≥ 1 ppb, 82%, 79% and 75% also had WLLS ≥ 1 ppb in F30S samples (*n* = 238), F3M samples (*n* = 237), and F6M samples (*n* = 132), respectively. (Table S4).

Some sites went from detect in FD samples to non-detect (ND) in flushed samples: F30S = 11%, F3M = 13%, and F6M = 15% (Table 3). Mean WLLs for samples with detectable lead (≥1 ppb) decreased with increased flushing (F30S: 2.11 ppb, *n* = 224; F3M: 2.04 ppb, *n* = 210; F6M: 2.00 ppb, *n* = 111). This indicates some value in flushing, however, even a six minute flush does not guarantee lower WLLs for all customers—5% of sites went from ND FD WLLs (<1 ppb) to detect (≥1 ppb) after flushing for 6 min (*n* = 218) (Table 3). Median WLLs for samples with detectable lead (≥1 ppb) remained the same after increased flushing (F30S: 2.00 ppb, *n* = 224; F3M: 2.00 ppb, *n* = 210; F6M: 2.00 ppb, *n* = 111). A small proportion of sites also went from ND FD WLLs (<1 ppb) to detect (≥1 ppb) after flushing for 30 s (7%, *n* = 374), and 3 min (6%, *n* = 372) (Table 3). For sites which had FD, F30S and F3M samples (*n* = 372), 28% had WLLs that increased by >1 ppb with a 30 s or 3 min flush.

### 3.2. Water Lead Levels and Flushing Efficacy Associated with Atypical Use Conditions

While not a planned part of the study, conditions arose which allowed us to evaluate the impact of a one-time 15-min utility flush on WLLs after lead service lines replacements at five residential sites. Lead service line replacements and construction are known to increase lead in water due to construction disturbances and galvanic corrosion for periods of weeks to years [19,30,38]. While over ten years have passed since Hurricane Katrina, the rebuilding process is still ongoing, and the city of New Orleans is still in the process of conducting 16,000 PLSLRs [48]. These conditions were evident during this sampling effort, as 43% of survey respondents reported there was street or side walk work on their block within the last year (*n* = 287). Five of our study participants contacted the city’s water utility after our testing to request removal of their lead service lines. All of the sites were sampled prior to, and after the line replacements and the utility or contractor 15-min post-replacement flush. Table S2 presents information about each home’s line replacement activity, occupancy status, and pre- and post-replacement sampling conditions and WLLs. While no definitive conclusions can be made about what impacted post-line replacement WLLs high WLLs (>EPA Pb AL of 15 ppb) existed a few days after line replacements in some occupied homes, after both a full line replacement (6 days later) and partial line replacement (Site 3, 1 and 2 days later), despite the post-replacement 15 min outdoor flush. Post-line replacement WLLs reached as high as 226 ppb one day after the partial line replacement (after a post-stagnation 30 s flush). However, by two weeks post-replacement, both of two homes sampled had low WLLs (<4 ppb), even after overnight stagnation.

### 3.3. Identifying Predictive Factors for Detectable WLLs

Select survey variables were evaluated using univariate and multivariate models to identify factors that may be correlated with detectable WLLs. Table S3 presents factors considered in univariate and multivariable mixed models and associated percentages of detectable WLLs in the FD samples. Table 4 presents the significant factors associated with detectable WLLs (≥1 ppb) after adjusting for flush time. A decreased likelihood of having detectable WLLs (≥1 ppb) was observed after both the 3- and 6-min flush time compared to FD samples without and with adjusting for number of occupants and era of home construction (odds ratio, OR = 0.68 and 0.58 with *p* < 0.001 and <0.001, respectively) (Table 4).

Besides flush time, the number of occupants and age of homes were significantly associated with detectable WLLs, after adjusting for flush time (Table 4). Lower occupancy homes and older homes (pre-1950) were associated with higher risk of detectable WLLs. The prevalence of detectable WLLs in FD samples decreased with occupancy: 1 occupant = 92%, 2–3 occupants = 67.6% and ≥4 occupants = 63.7% (Table S3). This trend is consistent with other prior studies, which show that less water use can increase water lead problems [55,56]. The prevalence of detectable WLLs in FD samples decreased in newer homes: Pre-1950 = 73.4%; Post-1950 = 48.5%, which is expected given increased WLLs content in plumbing of older homes. This same trend was observed for prevalence of detectable WLLs in older homes in F30S samples (*p* < 0.0001, *n* = 375), and F3M samples (*p* = 0.010, *n* = 373), but not for WLLs in F6M samples (*p* = 0.069, *n* = 218).

While pre-1950 homes are more likely to have lead service lines, the lack of information on their presence or absence at all sites limited our ability to evaluate the impact that lead service lines may have on WLLs and flushing efficacy. While there was significant difference in WLLs by neighborhood and zip code, with higher WLLs in older areas of the city which may have been more likely to have lead service lines (Kruskal-Wallis test, *p* < 0.05), the lack of random sampling, and the low sample size in many neighborhoods prevents any definitive conclusions about spatial variability in WLLs. While it is suspected that high WLLs associated with low occupancy homes may be due to reduced water usage, and hence greater water stagnation and Pb leaching, it was not possible to evaluate the association between number of occupants and water use due to the low number of sites reporting that data (*n* = 38).

### 3.4. Source Evaluation

**Figure 2:**
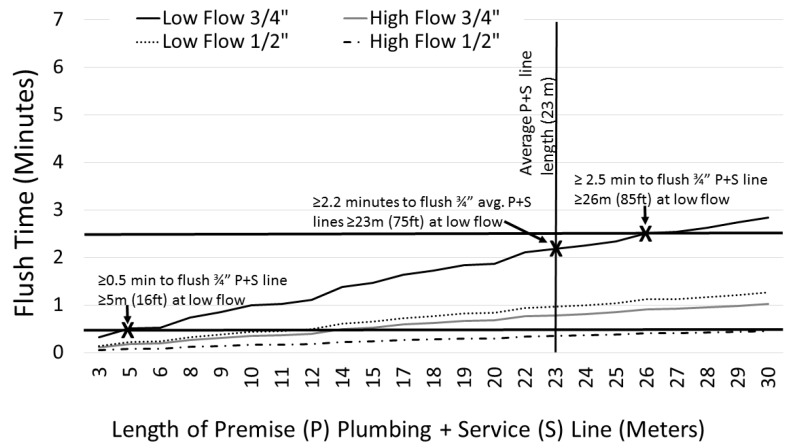
Estimated time to flush premise plumbing (P) and service line (S) (minutes) based on water flow rate (liters per minute), pipe diameter (3/4 or 1/2 inches) and survey-reported P + S length (meters) (*n* = −80) [**Note:** Low flow: 3.0 m per minute; High flow: 8.3 L per minute].

While lack of information on site plumbing materials limited our ability to ascertain the specific source of WLLs in New Orleans’ water system, the observation of peak WLLs in 30–45 s or 2 min flush samples at 49% of tested sites (*n* = 372), is consistent with the expectation that this flush time is very likely to capture water that was held inside the service lines [5,11]. An estimate of the volume of water and flush times required to purge the service lines of stagnant water was derived based on estimated flow rates, typical pipe diameters, and lengths of service lines and premise plumbing (*n* = 80). Figure S2 presents the distribution of line lengths (premise + service) reported by survey respondents (*n* = 80). The majority (75%) reported line lengths of 30 m of less; and one quarter of survey respondents measured premise plumbing plus service line lengths of >30 m. There were no significant differences in the lengths of service lines for pre- or post-1950 homes (*p* = 0.172). Figure 2 presents the estimated time to flush by total length of service lines and premise plumbing (¾ or ½ inches in diameter). Based on plumbing line length estimates presented in Figure 2, and an estimate that each 250-mL sample represents water in approximately 8 feet of piping; if a home has the average resident-reported premise + service pipe length of 75 feet (23 m) with a typical pipe diameter of ¾ inches, a flush time of 2.2 min would be required to purge any water sitting in the plumbing or service line over night, when water is run at low flow (3.0 L per minute). Under a different scenario in which residents run their taps at low flow and have an average reported pipe length of 30 m (premise + service line) approximately 3-min of flushing might be required. When the water is flushed at high flow (8.3 L per minute), the same system might be flushed in less than a minute (0.8 min). Given that study participants were instructed to collect water at normal to high flows, and the fact that most respondents had plumbing lengths of 30 m of less, it is likely that WLLs associated with service lines would often reach the tap by 30 s. This may explain why a peak WLL as high as 58 ppb was observed for in the F30S sample, which was from a single occupant home. More time is needed to fully flush the system as the length of plumbing increases, as the rate of water flow decreases, and as the diameter of the pipe decreases.

To identify the potential source of New Orleans’ water Pb (i.e., type of plumbing), correlations between WLLs and levels of common metals found in other plumbing materials were determined. If specific plumbing materials other than LSLs are associated with high WLLs, one would expect to see positive correlation between WLLs and metals in those alloys. For example, zinc may indicate the presence of galvanized water pipes or brass faucet fixtures; nickel may indicate the presence of brass faucet fixtures; iron may indicate the presence of iron water mains; copper may indicate the presence of copper pipe or brass faucet fixtures; tin may indicate the presence of leaded solder; chromium may indicate the presence of stainless steel and cadmium may indicate the presence of galvanized water pipes. No significant strong correlations were observed between any of the metals and WLLs for any of the flushed samples (all Spearman correlations < 0.3. Most samples had no detectable cadmium or tin. These results are consistent with lead from lead service lines, most of which are essentially pure lead, or possibly that many sources are contributing to WLLs in New Orleans water.

In Cartier et al. [57], the second consecutive sample was successfully used to confirm the presence of LSLs in 92% of homes for which lead service line presence was documented (at water temperatures above 17 °C). In that study, lead service line presence was considered confirmed if the second liter sample after a 15-min stagnation period exceeded 3 ppb. If such an approach were used it is necessary to validate WLL thresholds specific to the system and type of buildings sampled [40]. Given the lack of data to validate lead service line presence for New Orleans sites, we could not confirm a lead threshold for New Orleans that would enable accurate validation of the presence of lead service lines. However, we did observe for New Orleans homes with validated or reported lead service lines (*n* = 38), 37% had WLLs exceeding 3 ppb in FD samples (*n* = 38), as did 37% of F30S samples (*n* = 38), 40% of F3M samples (*n* = 38), and 32% of F6M samples (*n* = 28). In a similar vein, among the set of sites with validated or reported lead service lines combined with the set of “pre-1950” homes, which we use as an indicator of potential lead service line presence (*n* = 259), 27% of FD samples exceeded 3 ppb (*n* = 259), as did 30% of F30S samples (*n* = 258), 29% of F3M samples (*n* = 257), and 21% of F6M samples (*n* = 143). These data may lend support to the assumption that lead service line presence may lead to sustained WLLs.

Further investigations are needed to support the speculation that lead service lines are a primary risk contributor in New Orleans water. But together these data suggest that lead service lines may be a major contributor to New Orleans water Pb: (1) the sustained low WLLs throughout the New Orleans water systems (i.e., throughout all of the different sample types) (Figure 1); (2) the lack of strong significant correlations between WLLs and metals from other plumbing materials, (3) the occurrence of peak WLLs in flushed samples (Table 1); and (4) the significantly higher WLLs in homes more likely to have lead service lines (pre-1950 homes).

### 3.5. Comparison to Utility Compliance Sample Results and Evaluation of Sufficiency of FD Compliance Sampling

Sampling was not conducted as required under the Lead and Copper Rule, as samples were not collected exclusively in warm months (June–September), and sampled sites could not be verified as being high-risk (50% of sites with lead service lines). Thus WLL results are not be representative of required regulatory compliance samples. In the last utility-reported sampling season, the city water utility’s WLL data for post-stagnation first-draw regulatory compliance samples had a 90th percentile WLL of 7 ppb—only 1.6% of compliance samples exceeded the 15 ppb Pb AL (*n* = 60, S&WB 2017). Our results for WLLs in FD samples only are consistent with the utility’s compliance data, with a 90th percentile of 5.3 ppb; <1% of FD WLLs exceeded 15 ppb (*n* = 375, Table 1). A separate analysis was conducted to evaluate WLLs among FD samples, based on sites and samples that may meet regulatory sampling requirements (i.e., sites with reported or validated lead service lines and pre-1950 homes, and samples collected between June and September). The 90th percentile WLL remained within regulatory limits (5.4 ppb).

There is some debate about how representative the Lead and Copper Rule’s required FD compliance samples are of worst-case scenario exposures (i.e., highest WLLs). First draw samples are also frequently relied upon by many state lead poisoning prevention and school sampling programs to characterize potential risk [58,59]. Each sample type (FD, F30S, F3M and F6M) met AL requirements (90th percentile ≤ 15 ppb); though there were increases in the percent of sites exceeding the AL from 0.5% in FD samples, to 2.4% in F30S samples (Table 5). Even a small increase in the proportion of homes exceeding the AL could have an impact on regulatory compliance in cites that are on the borderline of Pb AL exceedances. While there were increases from the 90th percentile FD WLL value (5.4 ppb) after flushing for 30 s (6.0 ppb) and 3 min total (6.1 ppb) among tested sites, these increases were minimal (<1 ppb) (Table 1) and would not have exceeded the action level trigger, even if worse case flushed samples had been “counted” under the regulation.

### 3.6. Comparison of WLLs to Health Guidelines, Standards and Goals

To evaluate the public health relevance of results, WLLs were evaluated against existing health-based standards, guidelines or goals (Table 5). Twelve percent of samples had WLLs which exceeded the Food and Drug Administration’s Allowable Level (FDA AL) for Pb in bottled water (5 ppb) set in 1994 [53]; while only 2.7% of all samples at normal use occupied homes had WLLs which exceeded the World Health Organization’s provisional Pb Guidance Value (WHO GV) of 10 ppb set in 2011 [54]. The WHO GV is not entirely health-based, as other considerations, such as treatment performance and analytical achievability, were considered in GV derivation [54]—the WHO maintains that provisional guideline values are set for “contaminants for which calculated health-based values are not practically achievable” [60].

While the cumulative impact of low-dose chronic waterborne Pb exposure on fetuses, infants, children and pregnant women is uncertain, one study found that for every 1 µg/L increase in WLLs, childhood blood lead levels may increase by 35% after 150 days of exposure [10]. Recently, the American Academy of Pediatrics recommended a WLL limit for schools (AAP RL) of 1 ppb [52]. Overall, 60% of all samples from normal use occupied homes exceeded AAP’s RL for school water systems (Table 5, *n* = 1497). Given the fact that these low dose levels of Pb are widespread in New Orleans water, a large proportion of the city’s population of pregnant women and children may be at potential risk if they drink or cook with unfiltered tap water on a regular basis. Excluded from analyses were the WLL results of nine schools (*n* = 67 samples), of which, 27% exceeded 1 ppb. The percent of school samples with WLLs exceeding 1 ppb decreased with increased flushing: FD = 38%, *n* = 18; F30S = 28%, *n* = 18; F3M = 17%, *n* = 18; F6M = 10%, *n* = 10; however samples were collected only once during the day, after overnight stagnation.

Assuming that samples below the detection level (1 ppb) were true negatives, then samples exceeding the AAP recommended level for lead in school water (>1 ppb) also exceeded the EPA’s Maximum Contaminant Level Goal (MCLG) for Pb (0 ppb), the WLL that EPA considers to be safe [5]; and California EPA’s Public Health Goal of 0.2 ppb for Pb in water, which was decreased from 2.0 ppb in 2009 based on neuro-developmental effects of Pb for fetuses and children [61]. These results are pertinent, as 33% percent of our sample population reported having children less than six years of age (*n* = 376). The CDC is considering lowering the childhood blood reference value to 3.5 µg/dL [62]. The US EPA has released tentative results based on the Integrated Exposure Update and Biokinetic model (IEUBK), which estimate that WLLs of 3.8 ppb and 5.9 ppb could result in a 1% increase in the probability of a child (formula-fed infants and children 0–7 years of age, respectively) having a blood lead level of 3.5 µg/dL for families residing in pre-1950 homes with a high likelihood of having lead-based paint) [63]. Such home conditions are common in New Orleans—79% of this study’s respondents resided in pre-1950 homes. The percent of sampled sites with WLLs >3.8 ppb increased from 18% in FD samples, to 20% and 22% in F30S and F3M samples, respectively. This percentage declined to 14% in F6M samples. Similarly, the percent of sampled sites with WLLs > 5.9 ppb increased from 8% in FD samples, to 12% and 11% in F30S and F3M samples, respectively. This percentage declined to 6% in F6M samples. Thus, flushing according to prevailing exposure reduction guidelines (30 s to 2 min) may increase the likelihood of higher WLL exposures, and higher associated blood lead levels. Those performing longer flushes (F6M) could increase the likelihood of reducing their WLLs exposures and associated blood lead levels.

### 3.7. Evaluation of Potential Exposures to Lead in Water

Risks do not occur unless both a hazard and an exposure route to that hazard exists. To evaluate potential Pb exposure, survey respondents answered questions about water use habits, flushing practices, use of water treatment or filtration devices (survey in Supplementary Materials). Almost all respondents (93%) reported using unfiltered tap water for either cooking or drinking at some point in time (*n* = 277). Only 21% of survey respondents reported flushing water prior to use (*n* = 277). Of these respondents (*n* = 58), 48 reported their flush times—most flushed for half a minute or less (69%); 75% for 1 min or less; 92% for 2 min or less; and only 8% flushed for over 2 min. Peak WLLs for respondents reporting flushing occurred in: FHD samples for 40% of respondents; F30S samples for 29%; F3M samples for 29%; and F6M for 3%. While there was not widespread application of flushing guidelines among study participants, those who did flush water prior to use, may not be flushing long enough to see significant or substantial WLL decreases; and may also be inadvertently increasing exposures to WLLs (Table 5).

The greatest risks from exposures to waterborne Pb are expected for infants reliant on formula reconstituted with unfiltered water—15 respondents reported using unfiltered tap water to reconstitute baby formula (*n* = 129). WLLs for these study participants ranged from <1 to 11 ppb. Based on EPA’s preliminary IEUBK model estimates for formula-fed infants, WLLs of 11 ppb could result in elevated blood lead levels in formula-fed infants (>5 µg/dL) when exposures to other sources like soil Pb or Pb-based paint are taken into consideration [63]. When only water exposures are considered, WLLs of 11 ppb could result in formula-fed infant blood lead levels above the CDC-proposed Pb reference value (>3.5 µg/dL) [62], and/or a 1 µg/dL increase in geometric mean blood lead levels [63]. When cumulative exposures are considered, Ngueta et al. estimated that for every 1 µg/L increase in WLLs, childhood blood lead levels may increase by 35% after 150 days of exposure [10]. Among all samples, WLLs continued to exceed 1 ppb for the majority of all samples (60%, *n* = 1497); even after flushing for 30 s (61%, *n* = 375); for 2.5–3 min (58%, *n* = 373); and 5.5–6 min (52%, *n* = 218) (Table 1). These results suggest widespread exposure to WLLs of potential concern may be occurring in New Orleans for infants who are regularly fed formula reconstituted with unfiltered tap water.

## 4. Discussion

### 4.1. Flushing Efficacy and Practicality

Our results indicate that flushing taps according to prevailing utility and public health recommendations (i.e., for thirty seconds to two minutes) may not consistently reduce WLLs and associated exposures either significantly or substantially when applied in a city with lead service lines and at sites under normal use conditions (occupied residential sites with no prior line disruptions). In some cases, we observed that flushing for such short periods, especially after only 30–45 s, can actually increase WLLs, as predicted when lead service lines are present in a city [5,31,38]. It is generally agreed that first-draw samples may be more representative of Pb from the faucet and premise plumbing; while water flushed for 30 s to 2 min may be more representative of Pb in the service lines [64,65]. However, significant, but not always substantial, reductions in WLLs were observed after extended flushing (after 5.5–6 min). These samples are most likely representative of water held in the water main, which are generally not expected to contain Pb. When Pb is detected in samples collected after extended flushing, it may suggest the Pb is picked up during flow from premise plumbing or lead service lines. This can occur when there is Pb dissolution or particulate detachment from leaded plumbing [11,15,64].

In the aftermath of Flint, many school officials have been considering flushing as a routine water Pb exposure prevention measure. While the percent of residential and school samples with WLLs exceeding 1 ppb did decrease after extended flushing for 5.5 to 6 min, reductions in WLLs were not always substantial (>1 ppb). If the aim is to prevent childhood Pb exposure altogether, or at least reduce it to the minimal detectable levels (1 ppb) as recommended by the American Academy of Pediatricians, then New Orleans may require more proactive interventions to meet this goal, as over half of New Orleans residences and one in ten school samples collected still had detectable Pb (≥1 ppb) after extended flushing. In cases where extended flushing does reduce Pb to non-detectable levels, the question then becomes how frequently would it be needed (e.g., once a day, after certain time periods of water stagnation, prior to each use, etc.). Since sampling was only conducted at one point in time after a 6+ hour post-stagnation event, we could not verify that a one-time flush is sufficient to maintain low WLLs throughout the day. Some studies evaluating flushing at school taps suggest frequent flushes may be needed throughout the day, as waterborne lead can return to pre-flush levels within hours [66,67]. 

Prolonged and repeated flushing may also not be practical, cost-effective, or sustainable over the long term, especially in cities with declining water resources and/or rising water rates. In line with many states and utilities across the country, the state of Louisiana approved regular rate hikes in anticipation of water infrastructure repair needs—10% annually from 2013 to 2020 [68]. Yet, current water rates are already difficult for some New Orleans residents to afford. An estimated 10% of fiscal year 2015 New Orleans water customers were 30 or more days late in payment; and 19% of customer accounts were shut off for being unable to pay their bills [68]. New Orleans’ monthly residential water utility rate for fiscal year 2015 was $0.01 per gallon of water used or $69.20 per month (assuming an average monthly water usage of 9.24 hundred cubic feet or 6920 gallons) [67]. To put this into context the average fiscal year 2015 water rate for customers of public utilities in the U.S. was $0.005 per gallon or $36.39 for the same monthly water usage [68]. The same rate for Flint, Michigan, which has been touted as one of the highest water rates in the U.S., was $0.0167 per gallon or $115.56 for the same monthly water usage [69]. 

In cases where prolonged flushing could be effective for remediating high WLLs, such as after lead service line replacements, flush recommendations are not always consistent (Figure S4), and some recommendations (i.e., one-time 15 min high velocity flush) may not be effective for maintaining low WLLs over a long period of time as observed here after line-replacements (Table S2), and in prior studies [38,70,71,72]. It is widely acknowledged that sites with partial replacements may have higher WLLs; and may require more rigorous and regular flushing than normal-use residential sites under typical conditions [70]. However, utilities are not always required to promote flushing after all partial lead service line replacements, specifically, when replacements are conducted on a voluntary basis in Pb-compliant cities. More research is needed to evaluate how frequently flushing would need to be conducted to maintain low WLLs after a replacements. While we observed low WLLs (<4 ppb) in the two homes tested two weeks after replacements, authors of one study evaluating WLLs after simulating partial lead service line replacements in New Orleans, reported that intermittent flushing over a two week period was not long enough to stabilize WLLs [19]. These facts do not discount the benefits of more rigorous flushing protocols as an effective Pb remediation method for some systems when high WLLs are present. Improved remediation has been observed with higher velocity flushing (full open tap); continuous flushing (as opposed to intermittent flushing); increased flushing frequency and duration; and flushing at multiple taps [2,11,12,19,24]. But factors associated with maintaining low WLLs under such conditions, such as flushing rate, volume and frequency, may need to be determined on a case by case basis unless reliable trends can be documented under controlled conditions.

### 4.2. Regulatory Implications

Results underscore the importance of critically evaluating existing regulations in terms of their impact on reducing WLLs and Pb exposures. Mounting evidence, and US EPA assertions, also suggest that meeting the Lead and Copper Rule does not always guarantee public health protection [3,6,39,40,70,71,72,73,74,75,76,77]. One critical step in addressing a risk, is to identify the location of the hazard. The EPA recognized a decade ago the need to identify where lead service lines were installed, and henceforth required water systems to conduct audits of their service line materials. However, the cost and burden of this endeavor has resulted in a tolerated neglect of this responsibility by regulatory officials. Weaknesses are also evident in Lead and Copper Rule compliance sampling requirements [75]. For example, there are no stated requirements to include special-use sites like schools or homes with lead service line replacements from Lead and Copper Rule compliance sampling, as the intent of the Lead and Copper Rule is to evaluate worst-case WLLs under normal residential water use patterns. However, in line with many cities experiencing water infrastructure breakdowns or replacements, New Orleans has been conducting an unprecedented level of line replacements throughout the city, making these conditions and their associated risks more common. When such replacements were undertaken across the City of Flint, MI, all residents were notified of the risks and were provided free filters to remove lead for at least 6 months after replacements occurred [77]. As discussed previously, similar education and preventive measures are not required in cities compliant with the Lead and Copper Rule; thus, New Orleans’ water utility was not out of compliance when it failed to educate impacted residents of risks or preventive measures [72]. 

But even when cities meet Lead and Copper Rule Pb AL requirements, other weaknesses inherent in the regulation, that is the requirements to collect only first draw samples, could impact compliance status, as the highest WLLs in New Orleans water did not appear until after a 30 s or 2 min flush at most sites. While this change in sampling protocol would not have affected New Orleans’ compliance status, the difference we observed between FD and F30S samples in terms of the percent of samples exceeding the Pb AL (~2%) may be enough to impact compliance status for borderline systems with lead service lines. For example, the New Orleans residence exhibiting the highest WLL detected (58 ppb), had a FD WLL of only 7.8 ppb—the peak WLL did not occur until after the 30 s flush. Whether cities are compliant or not, there is always a risk of Pb exposure, especially in cities with lead service line, thus Lead and Copper Rule communication requirements should be revised to require regular consumer education on more evidence-based technologies for reducing exposures. Finally, over the years, health-based standards for Pb in blood have declined, but the Pb AL for drinking water has never received a similarly critical re-evaluation. As over one quarter of all samples collected from the nine New Orleans schools tested (*n* = 67 samples) exceeded American Academy of Pediatrician’s recommended WLL for schools (1 ppb) (Table 5), the Pb AL should be reconsidered in light of low dose Pb impacts on vulnerable populations; or a health-based trigger level should be developed.

### 4.3. Public Health and Risk Communication Implications

Infants, children and pregnant and lactating women are the most vulnerable populations. For these populations, the U.S. CDC [62,78] and National Toxicology Program (NTP) [1] have asserted that there is no safe level of Pb exposure. The neurotoxicity of very low blood lead levels on the developing fetal and neonatal brain have been widely acknowledged to be associated with adverse behavioral and cognitive effects [1]. Drinking water however, has frequently been overlooked as a potential source of Pb exposure in investigations of lead poisoning cases; despite the fact that EPA models indicate it can be the main contributor to infant blood lead levels [63], and it has been associated with adverse health impacts at the population-level [78,79].Yet, despite these facts and the weaknesses in the Lead and Copper Rule [75,76], the CDC still recommends no water testing is needed in the homes of a lead-poisoned child if other sources of high Pb were found in the home, if residents are not on private well water, and if the city’s water meets Lead and Copper Rule’s Pb AL requirements [80]. As such, public health officials may not have not been monitoring WLLs in the homes of lead-poisoned children, or educating impacted families about lead in water issues. Changes in public health policies should be made to ensure that CDC goals for preventing childhood Pb exposures are met [75,81]. Environmental monitoring of WLLs for the purpose of exposure assessment could be targeted to lead-poisoned children residing in cities known to have lead service lines—especially older or low-occupancy homes—risk factors which have been identified in this and prior studies [55,56]. Older homes are commonly identified as most likely to have lead service lines based on nationwide utility information [68]; and homes with low occupancy are hypothesized to have lower water use rates, more water stagnation, less buildup of corrosion control scale, less flushing out of particulates, and higher WLLs [55,56]. Until better condition-specific, evidence-based flush recommendations can be developed, public health officials, educators, water engineers and utility operators should work together to design communication strategies and consistent risk reduction messaging that promote evidence-based solutions, even when water systems are in compliance with lead-related regulations. Outreach that is only conducted during non-compliant periods may pose a risk to unsuspecting water consumers, especially if water lines are disturbed or replaced, as suggested in an investigation of New Orleans’ own water utility health communication practices [72]. In acknowledgement of this issue, the US EPA’s Lead and Copper Rule (LCR) Working Group recommended to US EPA officials in 2015, that the Consumer Confidence Reporting Rule be revised to exclude the currently required messaging: “When your water has been sitting for several hours, you can minimize the potential for lead exposure by flushing your tap for 30 s to 2 min before using water for drinking or cooking” [71]. Homogenized exposure reduction and lead remediation guidelines are always susceptible to error, given the wide variability that can exist between buildings, e.g., in pipe age, lengths, materials, and diameters; scale buildup; and home occupancy and water use. Promotion of these practices need to be reconsidered as other more effective, evidence-based, low-cost technologies, such as NSF-certified faucet mount filtration devices, are now widely available [82]. Finally, greater effort should also be expended on motivating and enabling proactive evidence-based solutions. Communication strategies and risk reduction messaging should translate current science about low dose Pb impacts on child and reproductive health to motivate proactive health-protective behaviors; and instruct residents in the correct selection, implementation and maintenance of NSF-certified filters. More research is needed to field test cost-effective household water filtration systems; evaluate these interventions’ likelihood for reducing chronic exposures to low dose waterborne Pb and associated blood lead levels; and measure the short- and long-term health impacts of chronic cumulative exposure to low dose waterborne Pb. If the intention is to prevent lead exposure, empowering individuals with the knowledge needed to motivate and support implementation of evidence-based household water treatment technologies should be paramount.

### 4.4. Study Limitations

This study could not answer the questions of what factors are critical to the efficacy of flushing (i.e., what impacts do water quality conditions or plumbing components have on flushing efficacy); and what are optimal flush conditions (flush time and frequency) under different water quality and plumbing scenarios. Specific conditions that could increase the risk of random Pb spikes, in particular, the presence of lead service lines, could not be evaluated given the lack of resident knowledge and utility information on plumbing materials. This information gap also prevented us from targeting the highest risk homes as required under the Lead and Copper Rule (sites with lead service lines, or copper with Pb solder); which in turn prevented us from evaluating regulatory compliance. However, this study does highlight the fact that flushing can be an inconsistently effective lead exposure prevention measure even in compliant cities, if the purpose of the flushing is not to remediate high WLLs, but rather to prevent chronic exposures to low level lead in vulnerable populations.

There were some weaknesses in the study design which could limit the generalizability of results. Sampling was conducted in only one city, yet each community water system has a unique set of water quality parameters which may have led to different conclusions. Sampling was also conducted on a small subset of New Orleans homes, and as demonstrated here, there can be significant variability in WLLs between sites within the same city. However, this fact also supports the conclusion of this study, which is that, in the absence of site-specific information on factors that can influence WLLs, a one-size-fits-all optimal flush time for sites within a city may be an unreliable exposure prevention measure. Sampling was also conducted at only one point in time, limiting our ability to evaluate the efficacy of a one-time flush for maintaining low WLLs throughout the day.

Convenience sampling may have introduced selection bias into both the WLL and survey results. New Orleans is comprised of a large proportion of minorities (59% African-Americans); and residents with low household incomes (38% make < $25,000); and only 36% have a college education or higher [83]. However, study participants were primarily Caucasian (75%), with incomes ≥ $75,000 (53%), and with college or graduate level educations (90%) (Table S3, Supplementary Materials). This may be due to under-coverage due to convenience sampling, and voluntary response bias. Non-response bias may not be a major concern. Of those eligible households (*n* = 405), 376 water kits were returned (93% response rate). Of eligible house with the valid water kits (*n* = 376), 96% of eligible participants returned the survey. It is also important to note that the water lead levels (WLLs) of the first draw cold water samples were similar between those responding to the survey (*n* = 361), and those who didn’t (*n* = 15) (the Wilcoxon rank-sum test *p*-value = 0.845). The medians (25% and 75%) of first-draw WLLs are 1.4 (0.5–2.95) ppb and 1.3 (0.5–2.6) ppb for those with and without the questionnaire.

Water samples were also collected by study participants, with no way to verify that samples were collected properly. Given the logistical difficulty in collecting post-stagnation water samples, utility compliance samples are also collected by residents. This may have resulted in some misclassification, but it also provided the advantage of generating samples from a large number of sites.

Finally, as this study presents environmental monitoring data for waterborne lead levels that were collected from each site at only one point in time; it did not provide a complete characterization of personal exposures to lead in water for the study participants. A prospective study engaged in the ongoing collection of biological data, as well as collection of data on other environmental lead hazards (e.g., soil, dust, paint, food, etc.) is essential to characterize true exposures and associated adverse health outcomes.

## 5. Conclusions

Overall New Orleans’ WLLs at occupied residential sites were typically low (≤5 ppb); however, low-dose detectable waterborne Pb is widespread across the city, and was observed to reach as high as 58 ppb. The majority of residential sites (>50%) exceeded health-based standards for children. Health standard exceedances are a concern as just over nine out of ten survey respondents reported either drinking or cooking with unfiltered tap water at some point in time. The sustained low WLLs throughout the New Orleans water system, the lack of strong correlations between WLLs and other metals, and the occurrence of peak WLLs in flushed samples, may indicate that lead service lines are a major contributor to New Orleans’ WLLs. Older homes (pre-1950) and low occupancy homes had significantly higher risk of detectable WLLs (≥1 ppb); and should be prioritized for outreach, monitoring and intervention, when lead service lines are present. While flushing taps according to prevailing guidelines (for 30 s to 2 min) may reduce WLLs for some homes, over half the tested homes had peak WLLS after the 30 s or 2 min flush, thus these recommendations may inadvertently increase exposures. Significant declines in WLLs were only seen after extended flushing (6 min), but these changes were not substantial (<1 ppb); and over half the extended flush samples had detectable lead. While flushing may not be consistently effective for reducing exposure, it is effective for remediating water with high particulate WLLs, but extended and more rigorous flushing may need to be repeated regularly for an indeterminate time period. If the aim of public health professionals is to prevent childhood Pb exposure, more effective interventions, like certified water filters, may be needed.

## Figures and Tables

**Table 1:** Distribution of post-stagnation water lead levels (WLLs) (ppb) under normal use conditions (New Orleans, LA, USA, 2015–2017).

Sample Type	N	Median WLL	Mean WLL	SD	25th Percentile WLL ^a^	75th Percentile WLL	90th Percentile WLL	Max WLL	% Detectable (≥1 ppb)
FD	375	1.4	2.3	2.5	0.5	2.9	5.3	16.5	65.3
FDH	156	1.3	2.2	2.7	0.5	2.4	4.4	17.8	60.3
F30S	375	1.7	2.9	5.0	0.5	3.2	6.0	58.1	61.3
F3M	373	1.4	2.5	3.0	0.5	3.2	6.1	22.1	58.2
F6M	218	1.1	1.9	2.1	0.5	2.3	4.2	11.9	52.3
All	1497	1.4	2.4	3.4	0.5	2.9	5.6	58.1	60.1

**Note:**^a^ Samples with WLLs below the reporting level were assigned a value of half the reporting limit or 0.5 ppb. **Key**: FD: first draw cold sample; F30S: flushing cold water for 30–45 s; F3M: flushing cold water for 2.5–3 min; F6M: flushing cold water for 5.5–6 min; FDH: first draw hot sample; WLL: water lead level; SD: Standard Deviation.

**Table 2:** Change in water lead levels (WLLs) after flushing (vs. FD WLLs) (ppb).

Samples ^a^	N	Median (25%, 75%)	Mean ± SD	Min	Max	90th Percentile	*p*-Value ^b^
F30S vs. FD	374	0 (−0.4, 0.6)	0.6 ± 4.2	−9.4	50.2	1.7	0.040
F3M vs. FD	372	0 (−0.5, 0.6)	0.2 ± 2.1	−12.5	15.4	2.1	0.219
F6M vs. FD	218	0 (−0.6, 0)	−0.2 ± 1.4	−5.8	7.5	0.7	<0.001
FDH vs. FD	155	−0.1 (−0.9, 0)	−0.4 ± 2.4	−12.9	12.1	0.7	<0.001

**^a^** FD: first draw cold sample; F30S: flushing cold water for 30–45 s; F3M: flushing cold water for 2.5–3 min; F6M: flushing cold water for 5.5–6 min; FDH: first draw hot sample; ^b^ Compare with FD based on the Wilcoxon signed-rank test.

**Table 3:** Flushing effectiveness based on reaching non-detectable water lead levels (WLLs) (ND: <1 ppb).

Parameter	F30S (%)	F3M (%)	F6M (%)
Detect in FD to ND	42 (11%)	50 (13%)	33 (15%)
ND in FD to detect	28 (7%)	23 (6%)	12 (5%)
No change (<1 ppb difference)	304 (81%)	299 (81%)	173 (80%)
Total n of sample type	374	372	218

**Key:** FD: first draw cold sample; F30S: flushing cold water for 30–45 s; F3M: flushing cold water for 2.5–3 min; F6M: flushing cold water for 5.5–6 min; ND: Non-detect (<1 ppb).

**Table 4:** Factors associated with percent of homes with detectable water lead level (WLL) (≥1 ppb) ^a^.

Effect	Univariate Model (*n* = 376)		Multivariable Model (*n* = 325)	
	OR (95% CI) ^b^	*p*-Value	OR (95% CI) ^b^	*p*-Value
**Flush time (min)**				
0	Reference		Reference	
0.5	0.82 (0.65–1.02)	0.079	0.78 (0.60–1.00)	0.053
3	**0.70 (0.58–0.85)**	**<0.001**	**0.68 (0.54–0.84)**	**<0.001**
6	**0.61 (0.50–0.74)**	**<0.001**	**0.58 (0.47–0.72)**	**<0.001**
**Occupants**				
0–1	-	-	Reference	
2–3			**0.26 (0.09–0.74)**	**0.012**
≥4			**0.20 (0.07–0.56)**	**0.003**
**Era build**	-	-		
Post-1950			Reference	
Pre-1950			**2.95 (1.80, 4.83)**	**<0.001**
Unknown			1.25 (0.60–2.61)	0.545

**Notes:**^a^ Based on the mixed model; bold: *p* < 0.05; ^b^ Odds ratio (95% confidence interval).

**Table 5:** Comparison of New Orleans WLLs (ppb) in normal use residential sites to standards.

Sample Type	N	% > AAP RL (1 ppb)	% > FDA AL (5 ppb)	% > WHO GV (10 ppb)	% > EPA’s AL (15 ppb)
FD	375	65.1	11.7	1.9	0.5
FDH	156	60.3	8.3	3.2	0.6
F30S	375	61.1	14.7	4.3	2.4
F3M	373	58.2	14.2	2.4	1.1
F6M	218	52.3	7.3	1.4	0.0
All	1497	60.0	12.1	2.7	1.1

AAP RL: American Academy of Pediatrics recommended water lead level for schools; FDA AL: United States Food and Drug Administration’s Allowable Levels of lead in bottled water; WHO GV: World Health Organization’s Guidance Value for lead in water; EPA AL: United States Environmental Protection Agency’s Action Level for lead in water; WLLs: Water lead levels.

## Supplementary Materials

The following are available online at http://www.mdpi.com/1660-4601/15/7/1537/s1. Table S1: New Orleans S&WB 2015 water quality data for finished water (after purification), Figure S1: The New Orleans, Louisiana Sewerage and Water Board’s 2015 Consumer Confidence Report presenting 2014 compliance sampling results. Figure S2: Percent of survey respondents by reported length of premise plumbing + service line measurements (meters) (*n* = 80). Figure S3: Distributions of the difference in water lead levels (WLLs) in cold water samples collected at first draw (FD) compared to WLLs in samples collected after various flush times. Table S2: Water lead levels (WLLs) in pre- and post-lead service lines replacement (LSLR) samples (*n* = 5. Table S3: Participant and household characteristics of respondents associated with detectable (≥1 ppb) water lead levels (WLLs). Table S4: Water lead levels (WLLs) in first draw vs. flushed cold samples. Figure S4. The New Orleans, Louisiana Sewerage and Water Board’s informational brochure: “Tips for reducing lead exposure from drinking water” (Source: NOLA S&WB’s 2016 Consumer Confidence Report). Survey for homes: Lead exposure assessment for drinking water study.
