# Supplementary material for: Effectiveness of Prevailing Flush Guidelines to Prevent Exposure to Lead in Tap Water
Source: Int J Environ Res Public Health. 2018 Jul 20;15(7):1537. doi: 10.3390/ijerph15071537 (PMC6068841; doi:10.3390/ijerph15071537)
Supplement: Supplementary file 1 [file ijerph-15-01537-s001.pdf]

Supplementary Materials

Katner et al 2018. Effectiveness of Prevailing Flush Guidelines to Prevent Water Lead Exposure in a Compliant City with Lead Service Lines

The following supplementary materials can be found in this document**:**

- **Table S1:** New Orleans 2015 water quality data for finished water (after purification)
- **Figure S1:** The New Orleans, Louisiana Sewerage and Water Board's 2015 Consumer Confidence Report presenting 2014 compliance sampling results.
- **Figure S2**: Percent of survey respondents by reported length of premise plumbing + service line measurements (meters)(n=80)
- **Figure S3:** Distributions of the difference in water lead levels (WLLs) in cold water samples collected at first draw (FD) compared to WLLs in samples collected after various flush times
- **Table S2:** Water lead levels (WLLs) in pre- and post- lead service lines replacement (LSLR) samples (n=5)
- **Table S3:** Participant and household characteristics of respondents associated with detectable (≥1ppb) water lead levels (WLLs)
- **Table S4:** Water lead levels (WLLs) in first draw vs flushed cold samples.
- **Figure S4:** The New Orleans Louisiana Sewerage and Water Board's informational brochure, "Tips for reducing lead exposure from drinking water" (Source: NOLA S&WB's 2016 Consumer Confidence Report)
- **Survey for homes:** Lead exposure assessment for drinking water study.

References referred to here can be found in the Reference section of the main paper.

Information about New Orleans Sewerage and Water Board Water Treatment System:

The New Orleans Sewerage and Water Board (S&WB) operates two water treatment plants- one on the East Bank and the other on the West Bank of New Orleans (NOLA). This study focused on homes served only by the S&WB's East Bank or Carrolton plant. The Carrolton plant provides an average of 135 million gallons of water per day to an estimated population of 286,603 (S&WB 2016). The plant uses a conventional treatment system to purify water from the Mississippi River. Ferric sulfate and polyelectrolyte is used for coagulation followed by flocculation and sedimentation. Chlorine, in the form of sodium hypochlorite, is used as the primary disinfectant and chloramines are used as the secondary disinfectant. Lime is used for corrosion control pH adjustment and sodium hexametaphosphate is added as a sequestrant. The final step are fluoridations followed by filtration through rapid gravity filters (sand and anthracite) (Black and Veatch 2016). The city's water quality parameters are presented in **Table S1, Supplementary Materials**.

Table S1. New Orleans S&WB 2015 water quality data for finished water (after purification)

Parameter	n	Min	Avg	Median	Max
Temperature (◦C)	588	9.5	22.4	23.4	33.4
Nephelometric turbidity (NTU)	588	0.07	0.12	0.13	0.79
pH	588	8.16	8.82	8.85	9.16
Free chlorine residual (ppm as Cl_2)	588	0.1	0.2	0.2	0.5
Polyphosphate (ppm)	240	0.10	0.33	0.32	0.59
Total alkalinity (ppm as $CaCO_3$)	588	82	126	127	172
Total chlorine (ppm)	588	0.7	3.0	3.2	4.2
Calcium (ppm)	588	36	55	51	66

Source: NOLA S&WB. Samples collected 1-1-2015 to 12-31-2015 from 11 points of entry to distribution system.

The New Orleans Sewerage and Water Board's Consumer Confidence Report Prior to Study Commencement (2015):

After EPA regulations on flush time recommendations were relaxed, the city's water utility, the Sewerage and Water Board, continued to promote the original flush recommendations from 2009 to 2015 [41, 46]. At the commencement of this study, the utility encouraged residents to flush their taps "for 30 seconds to 2 minutes before using water for drinking or cooking" daily under normal use conditions [41] (**Figure S1, Supplementary Materials**).

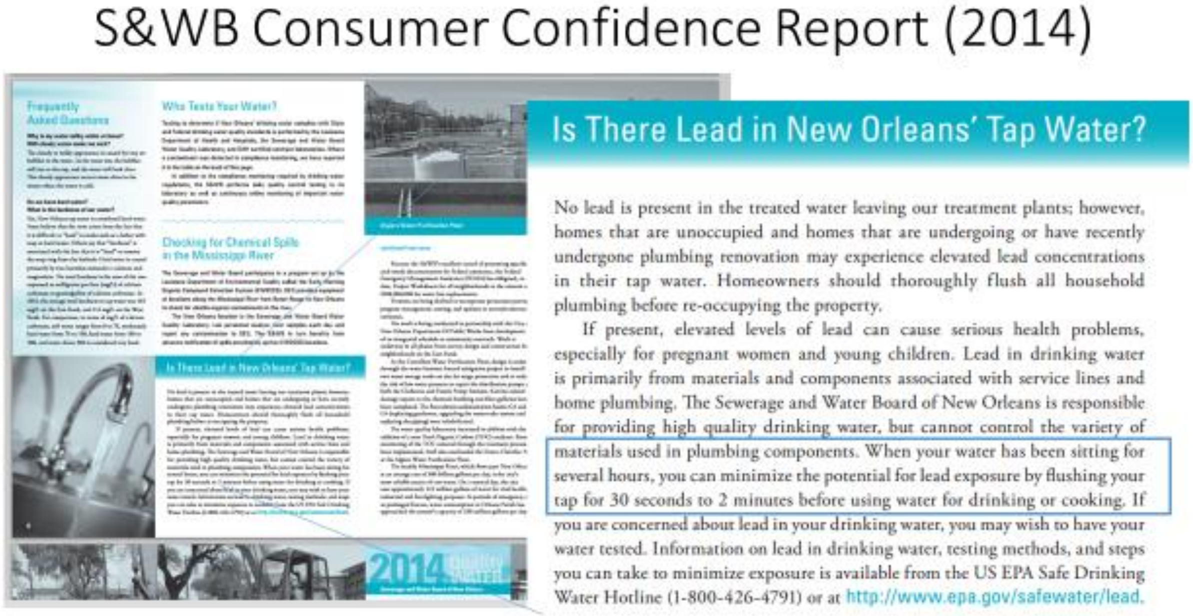

Figure S1. The New Orleans, Louisiana Sewerage and Water Board's 2015 Consumer Confidence Report presenting 2014 compliance sampling results. (Source: NOLA S&WB's 2015 Consumer Confidence Report, [41])

New Orleans, LA Study Participant Premise Plumbing and Service Line Lengths:

To determine the most probable location in the water distribution system or premise plumbing that each sample type may have been sitting during the stagnation period, an estimate of the volume of water and flush times required to purge the lines was derived based on estimated flow rates at low flow (3.0 liters per minute) and high flow (8.3 liters per minute); typical premise and service line pipe diameters; and survey respondent measurements of service lines and premise plumbing (**Figure S2, Supplementary Materials**). A 250-mL sample is estimated to represent water in approximately 2.4 meters (or 8 feet) of piping.

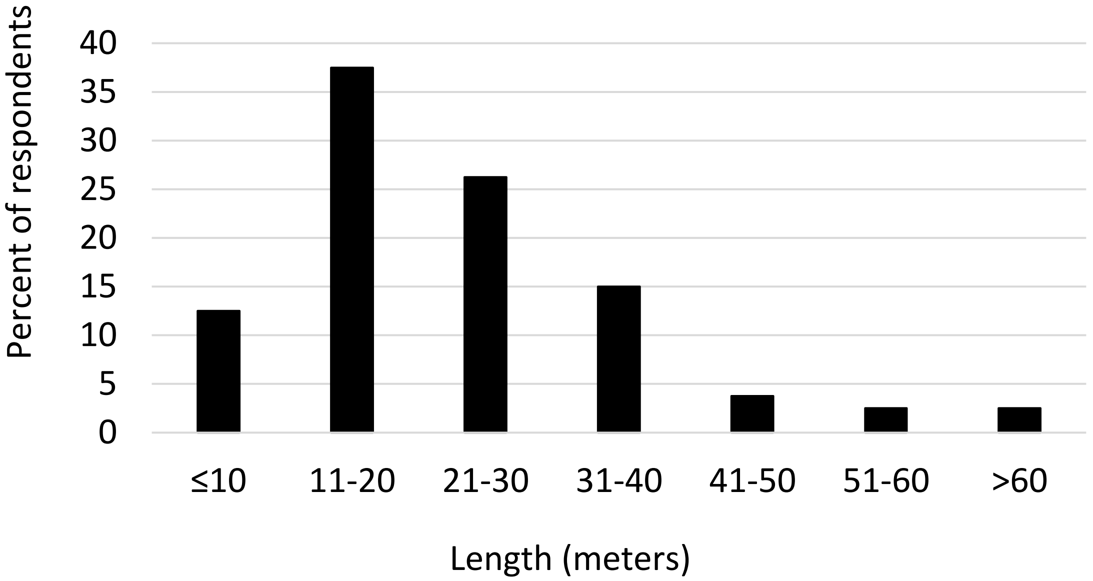

Figure S2. Percent of survey respondents by reported length of premise plumbing + service line measurements (meters)(n=80)

The lengths of water service lines and premise plumbing pipes were estimated based on resident measurements reported on returned surveys (**Figure S2, Supplementary Materials**). Residents were asked to measure the distance from the middle of the street to the water line as it enters the home (service line length) and the distance from where line enters home to the kitchen tap as measured along wall (premise plumbing). Researchers also derived google map measurements of potential service line lengths for all sites, based on measures taken from the center of the street to the front of the home in the satellite view of Google Maps using the distance and area tool.

Difference in Water Lead Levels in Flushed and First Draw Hot Samples Compared to First Draw Cold Water Samples:

The results of the WLL differences from FD to flushed samples (**Table 2, Figure S3**) demonstrate a small but significant increase (median=0 ppb and mean=0.6 ppb, p=0.04) in WLLs from FD to F30S sample. No significant change in WLLs was observed from FD to F3M samples (p=0.219). Small but significant declines in WLLs was observed in F6M (median=0 ppb and mean= -0.2 ppb) and FDH (median= -0.1 ppb and mean= -0.4 ppb) samples, compared to FD samples (**Table 2; Figure S3, Supplementary Materials**).

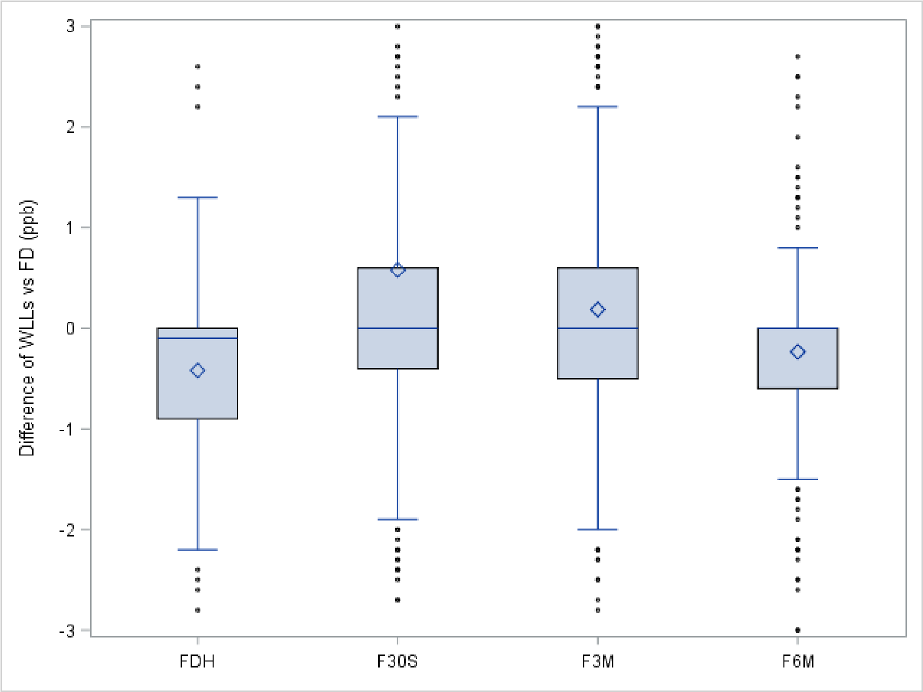

Figure S3. Distributions of the difference in water lead levels (WLLs) in cold water samples collected at first draw (FD) compared to WLLs in samples collected after various flush times

Key: FDH: first draw hot water; F30S: 30 second flush; F3M: 2.5-3 minute flush; and F6M: 5.5-6 minute flush samples. Diamonds inside box: mean value; line inside box: median value; bottom and top edges of box: 25th and 75th percentile; lower fence: 1.5 IQR (interquartile range) below 25th percentile; upper fence: 1.5 IQR above 75th percentile. **Notes:** Measures outside the range of -3 and 3 ppb were not shown. Refer to **Table 2** for the minimum and maximum values.

Water Lead Levels And Flushing Efficacy Associated With Atypical Use Conditions

While not a planned part of the study, conditions arose which allowed us to evaluate the impact of a one-time 15-minute utility flush on WLLs after lead service lines replacements at five residential sites. Lead service line replacements and construction are known to increase lead in water due to construction disturbances and galvanic corrosion for periods of weeks to years [19, 30, 38]. Five of our study participants contacted the city's water utility after our testing to request removal of their lead service lines. All but one of these residents received a partial lead service lines replacement (i.e., only the utility or customer side was replaced); while one had the full lead service lines replaced (from water main in the street to the home). All of the sites were sampled prior to, and after the line replacements and the utility or contractor 15-minute post-replacement flush. Only one of these homes was unoccupied due to ongoing home renovation work.

Table S2 shows sampling procedures and WLL results for each site- unfortunately, collection procedure for the samples varied from home to home. No definitive conclusions can be drawn from the post-line replacement samples due to the small sample size and variance in the sampling procedures. However, the persistent elevation in WLLs (exceeding the EPA AL) can be seen within the week after the line replacement in occupied homes, in both the full line replacement site (6 days later) and partial line replacement Site 3 (1-2 days later). Post-line replacement WLLs reached as high as 226 ppb one day after the partial line replacement (after a post-stagnation 30 second flush).

Table S2. Water lead levels (WLLs) in pre- and post- lead service lines replacement (LSLR) samples (n=5)

Site	**WLLs in pre-line replacement samples (ppb)**				**WLLs in post-line replacement samples (ppb)**				**Post-Line Replacement Samples** (after post-replacement 15-minute outdoor utility flush)
	FD	FDH	F30S	F3M	S1	S2	S3	S4	
Full LSLR	6.5	9.1	**49.2**[a]	12.6	**17.8**	3.0	2.8	NS	**S1: 6 days post-LSLR/OS- F30S**<br>S2: 2 weeks post-LSLR/OS- F30S<br>S3: 3 weeks post-LSLR/OS- F30S
Partial LSLR Site 1	5.0	2.6	5.8	9.8	0.5	0.5	0.5	0.5	2 weeks post-LSLR/OS:<br>S1: FD; S2: F30S; S3: F3M; S4: F6M
Partial LSLR Site 2	6.4	5.3	**24.7**	6.8	**16.8**	10.7	5.5	NS	**S1: Post-LSLR- FD (same day)**<br>S2: 2 days post-LSLR/OS- FD<br>S3: 3 days post-SLR/OS- FD
Partial LSLR Site 3	**30.0**	**151.9**	7.4	1.3	6.5	**225.8**	**61.8**	NS	S1: Post-LSLR- F30S<br>**S2: 1 day post-LSLR/OS- F30S**<br>**S3: 2 days post-LSLR, F30S**
Partial LSLR Site 4 (uninhabited)	**161.4**	NS	**26.6**	**283.5**	**64.5**	2.5	0.5	0.5	**Post-PLSLR (same day)**<br>**S1: FD;** S2: F30S; S3: F3M; S4: F6M

Note: [a] Bold WLLs exceeded the EPA AL of 15 ppb. **Key:** LSLR: lead service line replacement; WLLS: water lead levels; NS: no sample; FD: first draw sample; FDH: first draw hot water sample; F30s: sample collected after 30-34 second total flush; F3M: sample collected after 2.5-3 minute total flush; F6M: sample collected after 5.5-6 minute total flush; OS: overnight stagnation (6+ hour of stagnation prior to water collection); S#: Sample number.

These results suggest that rigorous extended flushing protocols may need to be repeated on a daily basis for an as yet indeterminate time period following line replacements. It is widely acknowledged that sites with partial replacements may have higher WLLs; and may require more rigorous and regular flushing than normal-use residential sites under typical conditions. [38, 70]. Post-replacement flush guidelines are not always consistent, and some guidelines (i.e., one-time 15 minute high velocity flush) may not be effective for maintaining low WLLs over a long period of time. In some circumstances, utilities are not required to promote flushing, such as after voluntary lead service line replacements in Pb-compliant cities.

When lead service line replacements are conducted in cities compliant with the Lead and Copper Rule, educating consumers about flushing is only required once a year, in the utility's annual Consumer Confidence Report for utility customers [3].

New Orleans has been undergoing extensive road work, including thousands of partial lead service line replacements [48]. For homes undergoing partial lead service line replacements, New Orleans officials recommend on their Roadwork website, that residents "Run cold water at a high flow at all of your faucets for at least 5 minutes each, one at a time, starting with the faucet closest to your water meter"; clean faucets aerators; and continue to flush for at least a month before using the water [71]. At the start of this study, this information was not consistently communicated nor readily available to New Orleans residents undergoing roadwork [72]. However, the persistent elevation in WLLs we observed days after the line replacements indicates that care should be taken to flush systems rigorously and regularly after line replacements (**Table S2, Supplementary Materials**).

The EPA's Science Advisory Board (SAB) stated that "the lack of mandatory water lead testing and homeowner education associated with voluntarily partial lead service line replacements suggests that in practice, voluntary replacement might be associated with greater exposure of the public to lead" [38]. The SAB recommends that utilities test the water and tell consumers to flush the lines "over a period of months" after a partial lead service line replacement; but concluded that while "line flushing appears to provide some benefit, the … time to realize the benefit (up to several weeks of flushing in the reviewed studies) likely precludes any practical implementation of this technique" [38]. Despite the general knowledge about the ineffectiveness and potential danger that partial lead service line replacements pose, they are still required by the Lead and Copper Rule when certain compliance conditions have not been met [3].

More research is needed to evaluate how frequently flushing would need to be conducted to maintain low WLLs after a partial lead service line replacement. One study simulated partial lead service line replacements in New Orleans, and observed that intermittent flushing over a two week period was not long enough to stabilize WLLs [19]. In keeping, previous studies suggest several weeks, months, or maybe years may be required to remediate increased WLL exposure after partial lead service line replacements [38, 74]. These facts do not discount the benefits of more rigorous flushing protocols as an effective Pb remediation method for some systems when high WLLs are present. Improved remediation has been observed with higher velocity flushing (full open tap); continuous flushing (as opposed to intermittent flushing); increased flushing frequency and duration; and flushing at multiple taps [9, 11-12, 19, 24].

However, residents should be alerted that when conditions are severe enough to warrant more rigorous flushing protocols, as observed here after partial lead service line replacements, exposures to high WLLs are always a possibility. Flushing can mobilize particulate-bound Pb throughout the plumbing system, which can then serve as a long-term source of acute Pb exposure. Even after flushing water for 10-25 minutes, some Flint homes still had high WLLs [14]- at least one Flint tap still contained WLLs exceeding 15 ppb (217-13,200 ppb) after a 26 minute flush [75]. This was likely due to the presence of highly unstable lead scales and the continuous sloughing of particulate lead during the time in which corrosion control was not used by Flint officials. Factors associated with maintaining low WLLs under such conditions, such as flushing frequency, must be determined on a case by case basis.

Associations between Participant and Site Characteristics and Detectable WLLs:

Participant and household characteristics (and associated minimum, mean, median and maximum WLLs) are presented in Table S3 (Supplementary Materials). Select survey variables were evaluated using univariate and multivariate models to identify factors that may be correlated with detectable WLLs. Table S3 presents factors considered in univariate and multivariable mixed models and associated percentages of detectable WLLs in the FD samples. Besides flush time, the number of occupants and age of homes were significantly associated with detectable WLLs, after adjusting for flush time (Table 4). Lower occupancy homes and older homes (pre-1950) were associated with higher risk of detectable WLLs. The prevalence of detectable WLLs in FD samples decreased with occupancy: 1 occupant = 92%, 2–3 occupants = 67.6% and ≥4 occupants = 63.7% (Table S3). This trend is consistent with other prior studies, which show that less water use can increase water lead problems [55,56]. The prevalence of detectable WLLs in FD samples decreased in newer homes: Pre-1950 = 73.4%; Post-1950 = 48.5%, which is expected given increased WLLs content in plumbing of older homes. This same trend was observed for prevalence of detectable WLLs in older homes in F30S samples ($p < 0.0001$, $n = 375$), and F3M samples ($p = 0.010$, $n = 373$), but not for WLLs in F6M samples ($p = 0.069$, $n = 218$).

Table S3. Participant & household characteristics of respondents & associations with detectable (≥1ppb) WLLs

Characteristics	n=361 N (%)	First Draw % of detectable WLLs (≥1ppb)	P-value[1]	OR (95% CI)[2]
**Number of occupants in household**				
1	25 (7.7)	92.0		
2-3	176 (54.2)	67.6	**0.01**	**0.27 (0.10, 0.73)**
≥4	124 (38.1)	63.7	**0.004**	**0.22 (0.08, 0.61)**
**Income**				
<50K	78 (26.4)	69.2		
51-74K	61 (20.7)	70.5	0.792	1.08 (0.59, 1.98)
≥75K	156 (52.9)	64.7	0.535	0.86 (0.53, 1.39)
**Race**				
Caucasian	257 (75.0)	70.8		
African-American or others	86 (25.1)	54.7	**0.013**	**0.59 (0.39, 0.89)**
**Education**				
High School and under	32 (10.0)	65.6		
College	114 (35.4)	64.9	0.677	0.86 (0.43, 1.73)
Graduate	176 (54.7)	69.9	0.974	1.01 (0.52, 1.98)
**Number of children <6 years old**				
0	188 (60.1)	69.2		
1	85 (27.2)	69.4	0.843	0.96 (0.61, 1.50)
≥2	40 (12.8)	55.0	0.268	0.72 (0.40, 1.29)
**Street sidewalk work in last year**				
No	167 (56.6)	66.5		
Yes	128 (43.4)	66.4	0.814	0.95 (0.64, 1.43)

Characteristics	n=361<br>N (%)	First Draw<br>% of detectable WLLs (≥1ppb)	P-value[1]	OR (95% CI)[2]
**Era home was built**				
Post-1950	66 (17.6)	48.5		
Pre-1950	248 (66.0)	73.4	**<0.001**	**3.01 (1.89, 4.81)**
Unknown	47 (13.0)	59.7	0.162	1.57 (0.83-2.96)
**Home type**				
Single family	247 (71.2)	67.5		
Multiple family/Apt complex	100 (28.8)	64.0	0.1516	0.74 (0.50, 1.12)
**Ownership**				
Own	276 (82.1)	69.2		
Rent	60 (17.9)	55.0	**0.024**	**0.58 (0.36, 0.93)**
**Water Usage**				
Monthly total (100 gallons)	45.9±28.1 (n=41)	73.2	-	-
Average daily (100 gallons)	1.8±2.4 (n=38)	71.1	-	-

Notes: "-"Sample size is too small (n<50) for solid modeling, Non-respondents were not included in the percentage and denominators; [1]Association with detectable (≥1ppb) vs. non-detectable lead level using mixed model adjusted for flush times. [2]Odds ratio (95% confidence interval)

Water Lead Levels (WLLs) in First Draw vs Flushed Cold Samples:

Table S4 presents the number of samples in each WLL category by sample type; and **Table 3** presents the number and percent of samples with a change in WLL detection or with insubstantial WLL changes (< 1 ppb), compared to FD WLLs. The majority of the households (80-81%) had no change in WLL (<1 ppb difference) in flushed samples compared to FD samples (**Table 3**). In general, most homes with FD WLLs below the reporting limit (<1 ppb) continued to have WLLs <1 ppb in flushed samples. Of sites with FD WLLs <1 ppb, 79%, 83% and 86% also had WLLs <1 ppb in F30S samples (n=136), F3M samples (n=135), and F6M samples (n=86), respectively (**Table S4**). Additionally, most homes with detectable FD WLLs (≥ 1 ppb) continued to have WLLs ≥ 1 ppb in flushed samples. Of sites with FD WLLS ≥1 ppb, 82%, 79% and 75% also had WLLS ≥1 ppb in F30S samples (n=238), F3M samples (n=237), and F6M samples (n=132), respectively. (**Table S4**).

Table S4. Water lead levels (WLLs) in first draw vs flushed cold samples.

FD WLL (ppb)	F30S WLL (ppb) (n)						F3M WLL (ppb) (n)						F6M WLL (ppb) (n)					
	<1	1-4.9	5-9.9	10-14.9	≥15	n	<1	1-4.9	5-9.9	10-14.9	≥15	n	<1	1-4.9	5-9.9	10-14.9	≥15	n
<1	108	27	0	0	1	136	112	22	1	0	0	135	74	12	0	0	0	86
1-4.9	41	134	15	2	4	196	48	127	18	1	1	195	33	75	3	0	0	111
5-9.9	1	6	22	3	3	35	1	10	20	3	1	35	0	8	9	2	0	19
10-14.9	0	2	2	1	0	5	1	1	2	0	1	5	0	0	1	0	0	1
>15	0	0	0	1	1	2	0	0	0	1	1	2	0	0	0	1	0	1
n	150	169	39	7	9	374	162	160	41	5	4	372	107	95	13	3	0	218

Key: FD: first draw cold sample; F30S: flushing cold water for 30-45 seconds; F3M: flushing cold water for 2.5-3 minutes; F6M: flushing cold water for 5.5-6 minutes.

Post-Study S&WB Risk Messages:

Homogenized exposure reduction and lead remediation guidelines are always susceptible to error, given the wide variability that can exist between buildings, e.g., in pipe age, lengths, materials, and diameters; scale buildup; and home occupancy and water use. Promotion of these practices need to be reconsidered as other more effective, evidence-based, low-cost technologies, such as NSF-certified faucet mount filtration devices, are now widely [82]. In acknowledgement of this issue, the US EPA's Lead and Copper Rule (LCR) Working Group recommended to US EPA officials in 2015, that the Consumer Confidence Reporting Rule be revised to exclude the currently required messaging: "When your water has been sitting for several hours, you can minimize the potential for lead exposure by flushing your tap for 30 seconds to 2 minutes before using water for drinking or cooking" [71]. Rather than promoting one-size-fits-all flush guidelines, greater effort should be expended on motivating and enabling proactive evidence-based solutions. Yet it was only after the preliminary release of our results in 2016 that S&WB revised their risk messaging and increased their flush guidelines to "30 seconds to 5 minutes"; however elsewhere in the same material, the messaging remained "30 seconds to 2 minutes"- conflicting messages which can increase confusion [84] (**Figure S4, Supplementary Material**).

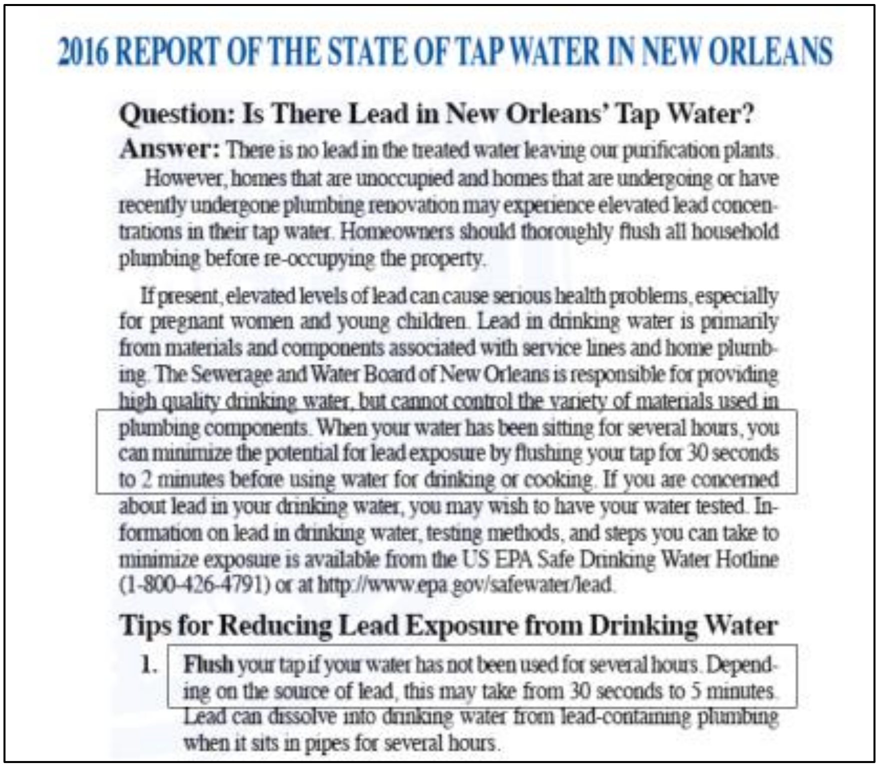

2016 REPORT OF THE STATE OF TAP WATER IN NEW ORLEANS

Question: Is There Lead in New Orleans' Tap Water?

Answer: There is no lead in the treated water leaving our purification plants.

However, homes that are unoccupied and homes that are undergoing or have recently undergone plumbing renovation may experience elevated lead concentrations in their tap water. Homeowners should thoroughly flush all household plumbing before re-occupying the property.

If present, elevated levels of lead can cause serious health problems, especially for pregnant women and young children. Lead in drinking water is primarily from materials and components associated with service lines and home plumbing. The Sewerage and Water Board of New Orleans is responsible for providing high quality drinking water, but cannot control the variety of materials used in plumbing components. When your water has been sitting for several hours, you can minimize the potential for lead exposure by flushing your tap for 30 seconds to 2 minutes before using water for drinking or cooking. If you are concerned about lead in your drinking water, you may wish to have your water tested. Information on lead in drinking water, testing methods, and steps you can take to minimize exposure is available from the US EPA Safe Drinking Water Hotline (1-800-426-4791) or at http://www.epa.gov/safewater/lead.

Tips for Reducing Lead Exposure from Drinking Water

1. **Flush** your tap if your water has not been used for several hours. Depending on the source of lead, this may take from 30 seconds to 5 minutes. Lead can dissolve into drinking water from lead-containing plumbing when it sits in pipes for several hours.

Figure S4. The New Orleans, Louisiana Sewerage and Water Board's informational brochure: "Tips for reducing lead exposure from drinking water" (Source: NOLA S&WB's 2016 Consumer Confidence Report)

The following pages present the participant survey:

LEAD EXPOSURE ASSESSMENT FOR DRINKING WATER STUDY

****

SURVEY FOR HOMES

Study participant________________________________ Date of home visit___________________________

Neighborhood__________________________________ Home visitor________________________________

Contact Information

1. Address:____

2. Contact Phone:___________________________ Contact email:__________________________________

3. Prefer to be contacted by: ☐ Phone ☐ Email

Water Tested

4. Tap to be tested: ☐ Kitchen ☐ Water-fountain ☐ Other: _____________________________

5. Floor of tap to be tested: ☐ 1st ☐ 2nd ☐3rd ☐ Other: _____________________________

Home Information

6. Do you own the home or rent? ☐ Own ☐ Rent

7. Type of building: ☐ Single family ☐ Double ☐ 4-Plex ☐ Larger Apartment/Condo

8. How old is the building/home? Year/Decade: _________ *OR* ☐ Pre1950 ☐ Post1950 ☐ Unknown

9. What year did you move into the home? ____

10. If no one currently lives in the home, how long has it been uninhabited? __________________________

11. Any new plumbing inside the home? ☐ Yes ☐ No ☐ Unknown

12. If known, when was the most recent plumbing repairs made (note location): ______________________

    ____

13. Do you know if any of these materials are in your plumbing? (check all that apply):
    ☐ Lead ☐ Plastic ☐ Galvanized metal ☐ Cast iron ☐ Copper ☐ Brass
    ☐ Other: ____
    ☐ Not sure about all plumbing materials in the home
    ☐ Do not know about any of the plumbing materials in the home

14. Do you have lead water service lines from the home to the street? ☐ Yes ☐ No ☐ Unknown
    a. If you don't know but want to find out you can go through the steps here to find out: http://apps.npr.org/find-lead-pipes-in-your-home/#intro

       Or scan the QR code with your smart phone or tablet.

b. Indicate if you went through this process to find out: ☐ Yes, I followed these steps ☐ No

15. Any partial or full replacement of water lines outside home? A full replacement is replacement of pipes from the home to the water main in the street. A partial replacement is just replacement of pipes from the meter to the main or from the meter to the home. ☐ None ☐ Partial ☐ Full ☐ Unknown

16. Any work on street or sidewalk in the last 6 months *within your block*? ☐ Yes ☐ No

17. Number of occupants in your building:____

18. Number of occupants under the age of 6 years in your home:___________________________________
    a. Are the children home-bound? ☐ Yes ☐ No ☐ Part-time
    b. Any there any other home-bound inhabitants? ☐ Yes ☐ No ☐ Part-time

Environmental Lead Levels

19. Ever tested the home for lead before? ☐ Yes ☐ No
    a. If yes, did you have lead anywhere? ☐ Yes ☐ No
    b. Where was the lead found?____
    c. Did you remediate or remove the lead source? ☐ Yes ☐ No ☐ Unknown

Water Use

20. Source of drinking water?
    ☐ Bottled water ☐ Filtration system ☐ Tap water (unfiltered) ☐ Pitcher filter
    ☐ Other: _________________________________

21. Do you ever use unfiltered water for cooking? ☐ Yes ☐ No

22. Do you ever use unfiltered water for drinking? ☐ Yes ☐ No

23. Do you ever use unfiltered *HOT* water for either drinking or cooking? ☐ Yes ☐ No

24. Do you flush your tap water before using? ☐ Yes ☐ No
    a. If yes, for how long?____

25. Does the tip of your kitchen faucet have a filter on it (aerator filter)? ☐ Yes ☐ No

26. If so, do you ever clean the filter at the end of your faucet? ☐ Yes ☐ No
    a. If yes how often? ☐ 1/week ☐ 1/month ☐ Other: _________________________

27. If you have infants, have you used unfiltered tap water for milk formula? ☐ Yes ☐ No

28. Average number of cups of *unfiltered* home tap water you drink per day:_____________________

29. If you have water filtration system, what kind?_____________________________________

30. Do you see any signs of corrosion in your plumbing, such as frequent leaks, rust-colored water or stained dishes or laundry? ☐ Yes ☐ No

31. Does your water have a bad (metallic) taste? ☐ Yes ☐ No

32. What is your reported water usage on your Sewerage and Water Board Bill for your last water bill?

See "THIS BILL" at top of your last S&WB statement:

a. Reading Date: ______________________________________

b. Water Usage (100 gal): _______________________________

c. Age Usage/Day (100 gal): ______________________________

Socio-demographic Information

We collect this information to make sure we are reaching low-income, minority populations who may be in need of outreach. This information will help us characterize our study population and redirect efforts if needed.

33. Race: ☐ Caucasian ☐ African-American ☐ Latin ☐ Asian ☐ Other______________________

34. Home Net Income: ☐ ≤$25k ☐ $26-50k ☐ $51-75k ☐ $76-100k ☐ >100k

35. Highest degree earned: ☐ Grade school ☐ High school ☐ College ☐ Graduate

Knowledge and Concerns

36. Have you ever been told about health hazards of lead, lead sources and ways to reduce exposures to lead by your doctor or other public health official? ☐ Yes ☐ No
    a. If yes, who was the source of this info? ____

37. Any lead-related issues or concerns? ____

    ____

    ____

Lead Poisoning

38. Have you ever had your child tested for lead? ☐ Yes ☐ No (jump to question 39)
    a. If so was his/her lead elevated (>5 ug/dL)? ☐ Yes ☐ No
    b. What was the age of the child and year of test?____________________________________
    c. If you know the source of exposure, what was it? ___________________________________
    d. Did you remove or remediate the source of exposure? ☐ Yes ☐ No
    e. If you tested the child's blood lead level again after you removed the source of exposure how did his/her blood lead level change? ☐ Went up ☐ Went down ☐ Stayed same

Home Measurements (feet) (if have time)

If you have time, please see the diagram on the following sheet and get measurements for your home's plumbing. This information may assist us in estimating optimal flush times for reducing people's exposure to lead.

39. Distance from shut-off valve or water meter to front of home (A):__________________

40. Distance from water main in middle of street to front of home (B):_________________

41. Distance of internal plumbing from front of home to tap to be tested:_______________
    (Measured along the wall from the front where pipe enters home to the tap)

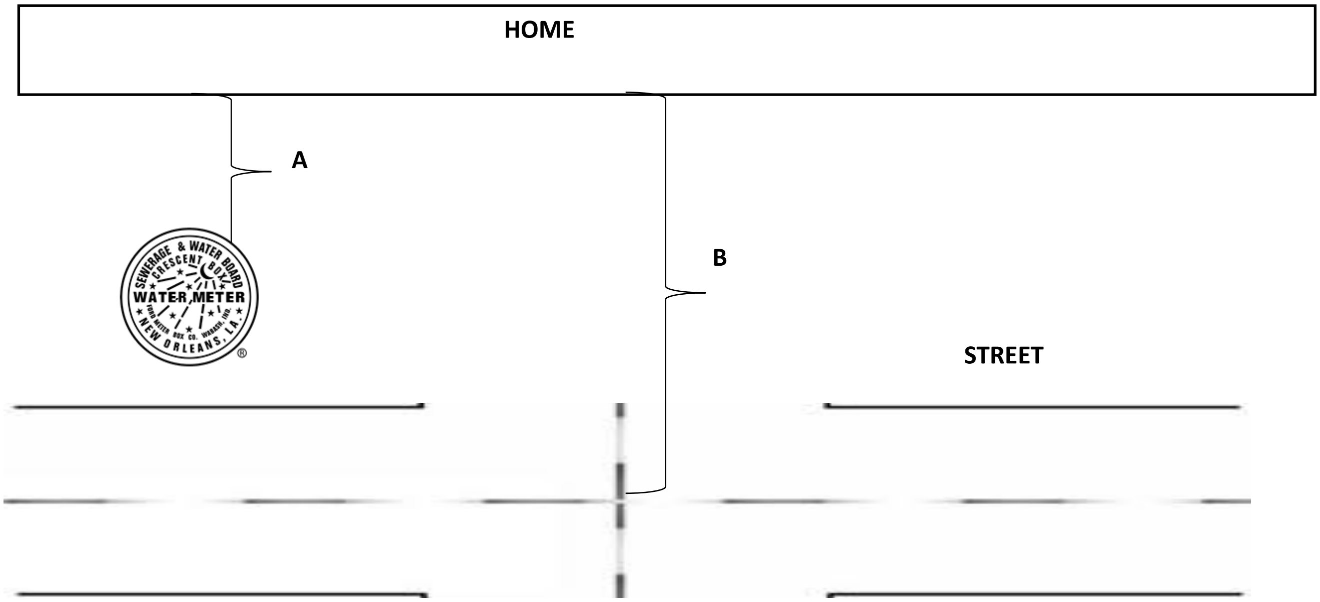

42. Finally, how did you find out about the study?
    ☐ LSUHSC letter ☐ Media ☐ Word of mouth

    Other: ____

Use this space for any additional comments or concerns.

Thank you for participating in this study.
We will contact you shortly as soon as the water test results are received,
and give you guidance on next steps if needed.
